# *Drosophila *olfactory local interneurons and projection neurons derive from a common neuroblast lineage specified by the *empty spiracles *gene

**DOI:** 10.1186/1749-8104-3-33

**Published:** 2008-12-03

**Authors:** Abhijit Das, Sonia Sen, Robert Lichtneckert, Ryuichi Okada, Kei Ito, Veronica Rodrigues, Heinrich Reichert

**Affiliations:** 1 Department of Biological Sciences, Tata Institute of Fundamental Research, Mumbai, India; 2 National Centre for Biological Sciences, Tata Institute of Fundamental Research, Bangalore, 560065, India; 3 Biozentrum, University of Basel, Basel, Switzerland; 4 Institute of Molecular and Cellular Biosciences, University of Tokyo, Tokyo, Japan; 5 Kagawa School of Pharmaceutical Sciences, Tokushima Bunri University, Sanuki, Japan

## Abstract

**Background:**

Encoding of olfactory information in insects occurs in the antennal lobe where the olfactory receptor neurons interact with projection neurons and local interneurons in a complex sensory processing circuitry. While several studies have addressed the developmental mechanisms involved in specification and connectivity of olfactory receptor neurons and projection neurons in *Drosophila*, the local interneurons are far less well understood.

**Results:**

In this study, we use genetic marking techniques combined with antibody labelling and neuroblast ablation to analyse lineage specific aspects of local interneuron development. We find that a large set of local interneurons labelled by the GAL4-*LN1 *(NP1227) and GAL4-*LN2 *(NP2426) lines arise from the lateral neuroblast, which has also been shown to generate uniglomerular projection neurons. Moreover, we find that a remarkable diversity of local interneuron cell types with different glomerular innervation patterns and neurotransmitter expression derives from this lineage. We analyse the birth order of these two distinct neuronal types by generating MARCM (mosaic analysis with a repressible cell marker) clones at different times during larval life. This analysis shows that local interneurons arise throughout the proliferative cycle of the lateral neuroblast beginning in the embryo, while uniglomerular projection neurons arise later during the second larval instar. The lateral neuroblast requires the function of the cephalic gap gene *empty spiracles *for the development of olfactory interneurons. In *empty spiracles *null mutant clones, most of the local interneurons and lateral projection neurons are lacking. These findings reveal similarities in the development of local interneurons and projection neurons in the olfactory system of *Drosophila*.

**Conclusion:**

We find that the lateral neuroblast of the deutocerebrum gives rise to a large and remarkably diverse set of local interneurons as well as to projection neurons in the antennal lobe. Moreover, we show that specific combinations of these two neuron types are produced in specific time windows in this neuroblast lineage. The development of both these cell types in this lineage requires the function of the *empty spiracles *gene.

## Background

**Figure 1:**
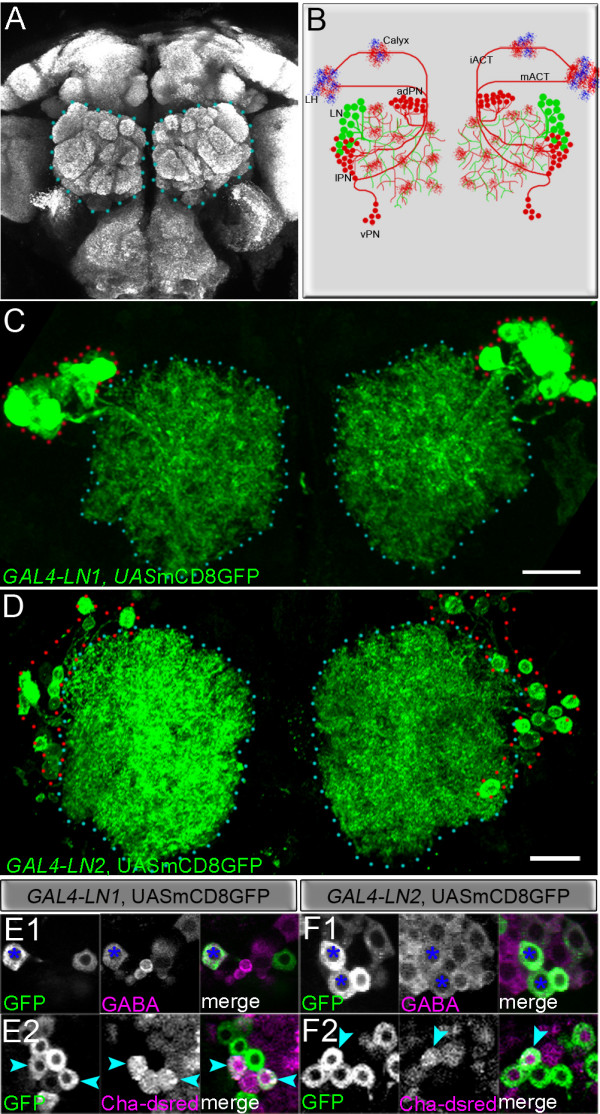
Architecture of the adult *Drosophila *olfactory circuit and local interneurons marked by GAL4-*LN1 *and GAL4-*LN2*. **(A) **Adult brain stained with mAbnc82, which recognizes presynaptic terminals. The antennal lobes are demarcated with blue dotted lines. **(B) **Schematic representation of olfactory interneurons. Note the three clusters of projection neurons (PNs; red) in anterodorsal (adPN), lateral (lPN) and ventral (vPN) locations and the single cluster of local interneurons (LNs; green) in the dorsolateral location. LNs ramify multiple glomeruli and PNs project from the antennal lobe to the calyx of the mushroom bodies and the lateral horn (LH). mACT, medial antennocerebral tract; iACT, inner antennocerebral tract. **(C, D) **Cell bodies of GAL4-*LN1 *(C) and GAL4-*LN2 *(D) are clustered lateral (encircled by red dots) to the lobe (encircled by blue dots). Scale bars, 20 μm. **(E-F) **Neurotransmitter identity of the LNs. Cell bodies of GAL4-*LN1*, UAS-mcD8::GFP (E1) and GAL4-*LN2*, UAS-mcD8::GFP (F1) were immunolabelled by antibodies to GABA (blue asterisks). A few cells expressing *Cha*-dsRed were detected (E2, F2; cyan arrowheads). Genotype in (F): GAL4-*LN2*, UAS-mCD8::GFP/*Cha*-dsRed and *Cha*-dsRed/+; GAL4-*LN1*, UAS-mCD8::GFP/+.

Antennal lobes, the insect counterpart of the vertebrate olfactory bulbs, are the primary centres for olfactory processing. They are subdivided into individual glomeruli, which are typical of primary olfactory systems in many animals (Figure 1A). Three principal populations of neurons form synapses in the glomerular neuropile [1]. Olfactory receptor neurons (ORNs) from the olfactory sense organs make synapses with two major types of olfactory interneurons in the antennal lobes, namely the projection neurons (PNs) and the local interneurons (LNs). The PNs receive excitatory input from ORNs and relay olfactory information from the glomeruli to higher brain centres such as the mushroom body and lateral horn. LNs are intrinsic interneurons, which, together with ORNs and PNs, establish a complex synaptic network in the antennal lobe characterised by diverse interglomerular connectivity patterns (Figure 1B).

The developmental mechanisms that give rise to ORN and PN circuitry have been studied in great detail in *Drosophila *[2-4]. In flies, as in mammals, precise neuronal circuitry is established by the ordered axonal projections of ORNs that express a given odorant receptor molecule type to specific target glomeruli in the antennal lobe [1,5,6]. In the antennal lobe, comparably precise circuitry is established by the PNs, many of which target their dendrites in a highly stereotyped manner to specific glomeruli [7-10]. The approximately 150 PNs in *Drosophila *derive from three deutocerebral neuroblasts, the anterodorsal neuroblast (adNb), the lateral neuroblast (lNb) and the ventral neuroblast (vNb). The dendritic targeting specificity of anterodorsal PNs is reported to be pre-specified by lineage and birth order [9]. Several intrinsic transcription factors as well as gradients of axonal guidance molecules are known to control this PN targeting process independent of ORN axons [11-14]. PN axons form spatially highly stereotyped terminal projections in the mushroom body and lateral horn according to the glomeruli that their dendrites innervate [15-19].

In contrast to studies on the development of ORNs and PNs, significantly less is known about the cellular and molecular mechanisms that control neurogenesis, process outgrowth and connectivity of the LNs. In *Drosophila*, there are thought to be on the order of 100 multiglomerular LNs in each antennal lobe [20]. There is a growing appreciation of the important functional role of LNs in the transformation of olfactory signals in the antennal lobe. LNs form an extensive network of inhibitory and excitatory synaptic connections with both PNs and ORNs, and these interconnections play central roles in olfactory feature extraction and in shaping odour-evoked activity patterns in the antennal lobe [21-25]. Some insight into the developmental origin of a subset of these olfactory LNs has been obtained by combining neuroblast ablation with GAL4 reporter labelling. These experiments suggest that the approximately 20 LNs labelled by the GH298 driver could derive from the lNb [20]. Most recently, while this article was under review, Lai and his colleagues [26] carried out an extensive clonal analysis to show that the lNb gives rise to a diverse population of cells, including the LNs, uniglomerular and multiglomerular PNs as well as neurons that innervate neuropile outside the antennal lobe.

In this study, we trace the development of the LNs that innervate the antennal lobe using mosaic analysis with a repressible cell marker (MARCM)-based genetic labelling and mutational techniques combined with antibody markers and neuroblast ablation. Our results support data from Lai *et al*. [26] indicating that LNs arise from the lNb, which also gives rise to the lateral PNs. We show that the LNs are born throughout the proliferative divisions of the lateral lineage and uniglomerular lateral PNs (lPNs) are generated during later divisions. Moreover, we observed a striking diversity in the innervation patterns of LNs. Finally, we demonstrate that this lineage requires the normal function of the cephalic gap gene *empty spiracles *(*ems*) for LN and lPN development. Our findings lay the groundwork for subsequent analysis of cell intrinsic and non-autonomous cues that could underlie the specification of LNs and PNs in the olfactory system of *Drosophila*.

## Results

### Developmental origin of LNs

To investigate the development of LNs, we first studied the expression patterns of a number of currently available GAL4 lines – GAL4-*NP1227 *(henceforth referred to as GAL4-*LN1*), GAL4-*NP2426 *(referred to as GAL4-*LN2*), *Krasavietz*-GAL4, GAL4-*KL78 *andGAL4-*KL107 *– which label populations of cells, including the olfactory LNs [24,25,27]. In these experiments, GAL4 was used to drive a UAS-mCD8::GFP reporter and the monoclonal antibody nc82 was used to highlight the olfactory glomeruli as well as other brain neuropiles. In all cases, the populations of LNs were recognised by their profuse arbors throughout the antennal lobe, which lacked projections outside the glomerular neuropiles (Figure 1C,D and Additional file 1B–D). The somata of the labelled cells with antennal lobe arbors were found clustered in a similar region lateral or dorsolateral to the antennal lobe (Figure 1 and Additional file 1).

To analyse the labelled cells further, we focused on the GAL4-*LN1 *and GAL4-*LN2 *lines. GAL4-*LN1 *labels LNs in a lateral cell body cluster of the antennal lobe (Figure 1C), while GAL4-*LN2 *labels a large number of LNs in this cluster and a few neurons in the ventral cell body cluster. The GAL4-*LN1 *and GAL4-*LN2 *lines have been characterized to mark a median of 18 (range 14-20) and 38 cells, respectively, in a largely non-overlapping manner with some amount of variability [25]. We subjected these strains to the mosaic analysis with a repressible cell marker (MARCM) technique to label single cells [28]. These single-cell clones confirmed that the labelled cells were indeed olfactory LNs. Thus, all the cells labelled with GAL4-*LN1 *as well as the lateral population labelled with GAL4-*LN2 *had a single neurite, which extended from the cell body into the glomerular neuropile where it arborised widely in several glomeruli. With the exception of this single cell body neurite, no processes were found outside of the glomerular neuropile. The labelled LNs differed in their putative neurotransmitter as assayed by immunocytochemistry. As expected, in the cell populations labelled by GAL4-*LN1 *or GAL4-*LN2 *many cells showed immunoreactivity characteristic for GABA-ergic transmission (Figure 1E1,F1), but there were also cells that were indicative of cholinergic transmission (Figure 1E2,F2). Cholinergic LNs innervating the antennal lobe have been demonstrated before [24].

The consistent location of the somata of the labelled LNs lateral to the antennal lobe suggests that most LNs might derive from one or more Nbs located in the same general region. To investigate this, we carried out Nb ablation experiments comparable to those performed by Stocker *et al*. [20], but using the GAL4-*LN1 *and GAL4-*LN2 *lines together with a UAS-mCD8::GFP reporter and the mAbnc82 neuropile labelling. In these experiments, the DNA-synthesis inhibitor hydroxyurea (HU) was fed to larvae at 0–4 h after larval hatching (ALH). At this stage only five pairs of Nbs, the four mushroom body Nbs and a lateral Nb, are reported to be dividing and are thus prone to ablation by HU [20,29-31]. In non-treated control adults, GAL4-*LN1 *and GAL4-*LN2 *lines drive expression in approximately 20 and 40 cells, respectively (Additional file 2A,C). In all cases, these cells had widespread multiglomerular arbors in the antennal lobe as expected for olfactory LNs.

In HU-treated animals, the antennal lobes were often reduced in size and composed of distinctly smaller glomeruli (Additional file 2B,D). The limiting dosage of HU used in our experiments produced some brains in which the effects were restricted to one side of the brain (yellow dotted lines in Additional file 2), allowing comparison with an unaffected antennal lobe (blue dotted lines in Additional file 2). Importantly, whenever a size-reduced antennal lobe was recovered, we observed a near complete absence of labelled LNs; both labelled somata and arborisations in the affected lobe were missing in GAL4-*LN1 *as well as in GAL4-*LN2 *lines. Occasionally, we recovered size-reduced antennal lobes associated with one or two labelled cell bodies, which were probably born before the HU treatment killed the lNb.

These findings suggest that the approximately 60 olfactory LNs labelled by GAL4-*LN1 *and the lateral population of GAL4-*LN2 *may derive from the lNb. Together with the earlier results of Stocker *et al*. [20], these data imply that a large proportion of the olfactory LNs could derive from this lateral lineage.

### LNs share a Nb lineage with the lateral cluster of PNs

**Figure 2:**
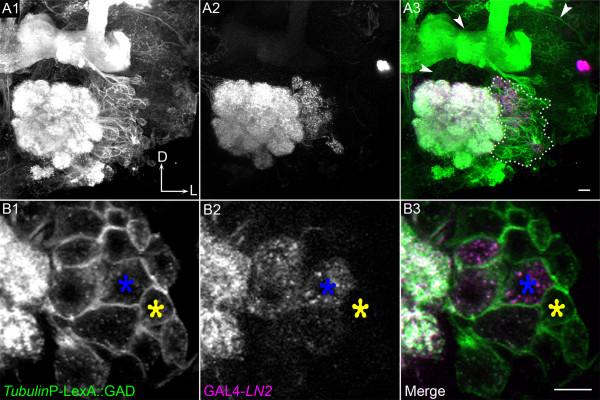
Local interneurons (LNs) arise from the lateral neuroblast (lNb) lineage. **(A) **The entire lNb clone is visualised by *Tub*-LexA::GAD; LexAop-rCD2GFP (A1). (A2) The same cluster includes all GAL4-*LN2 *marked cells. In the merge (A3) the entire lateral cluster is encircled by a white dotted line and the higher centre projections of the PNs from the lateral cluster are indicated by arrowheads. D, dorsal; L, lateral. **(B) **Higher magnification images of single section of the cells in (A) showing *Tub*-LexA::GAD; LexAop-rCD2::GFP cells within a Nb clone (B1). The clone also contains GAL4-*LN2 *expressing cells (B2) marked with blue asterisks. Some cells were not labeled by GAL4-*LN2 *(shown by yellow asterisks). Scale bars, 10 μm. Genotype: GAL4-*LN2/Tub*-LexA::GAD; FRTG13, hsFLP, *Tub*-GAL80/FRTG13, UAS-mCD8, LexA-oprCD2::GFP.

If many LNs do indeed derive from the lNb, they would belong to the same lineage as the lPNs that are also generated from these progenitors [9,20]. Hence, the same brain Nb would generate two sets of neuronal progeny that are markedly different in cytoarchitecture, connectivity and function. To investigate this and to determine the proliferation pattern and lineage relationships for LNs and lPNs, we carried out two series of dual expression-control MARCM experiments [32]. Clones were induced 0–4 h ALH and recovered in the adult. In the first series of experiments, GAL4-*LN2 *and ubiquitously expressed *tub*-LexA::GAD were used as drivers (LN2 dual MARCM), thus allowing simultaneous differential labelling of the GAL4-*LN2 *expressing LNs (via GAL4-*LN2*-driven UAS-mCD8) and of all cells in a Nb clone (via *tub*-LexA::GAD-driven lexAop-rCD2::GFP). All of the double-labelled Nb clones recovered in these experiments had similar features. The *tub*-LexA::GAD-driven marker expression labelled an entire Nb clone consisting of a large number of cell bodies (183 ± 25; N = 10) located lateral to the antennal lobe. The antennal lobe neuropile was also intensively labelled, indicating that these cells extend numerous processes into the glomeruli (Figure 2A). The overall morphology of these *tubulin*-labelled clones corresponds to that reported for the postembryonic lNb lineage [32,33]. By contrast, the GAL4-*LN2*-driven marker labelled only a subset of cells (approximately 30) in the Nb clone (Figure 2B). These *LN2*-labelled LNs had cell bodies that were clustered together in the dorsolateral region of the Nb clone next to the antennal lobe (Figure 2B2). The processes of the labelled LNs ramified extensively within the glomeruli, as expected, and did not project out of the antennal lobe.

Some of the cells within the *tub*-GFP-labelled Nb clone appeared to be PNs given that a labelled axon bundle was seen projecting from the labelled antennal lobe towards the higher brain centres (white arrowheads in Figure 2A3). In order to investigate this, we carried out a second series of dual expression-control MARCM experiments in which GAL4-*GH146 *and *tub*-LexA::GAD were used as drivers (GH146 dual MARCM) in order to differentially label *GH146*-expressing PNs (via GAL4-*GH146*-driven UAS-mCD8) together with all cells in the Nb clone (via *tub*-LexA::GAD-driven lexAop-rCD2::GFP). As expected, three spatially distinct clusters of double-labelled clones were recovered corresponding to the lineages of the adNb, lNb and vNb [9]. We restricted our analysis to the cluster of labelled cells that represents the lateral neuroblast lineage.

**Figure 3:**
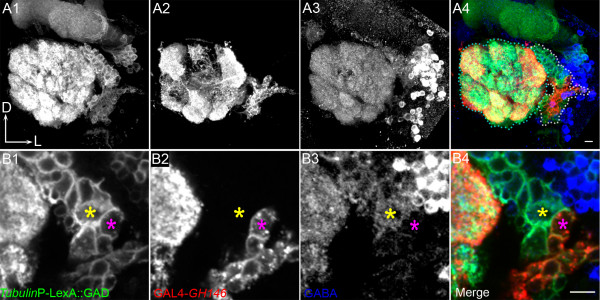
Projection neurons (PNs) arise together with local interneurons (LNs) from the lateral neuroblast (lNb). **(A) **The entire lNb clone is visualised by *tub*-LexA::GAD; LexAop-rCD2::GFP (A1). D, dorsal; L, lateral. (A2) GAL4-*GH146 *cells within the lateral cluster; (A3) GABA staining; (A4) merge. **(B) **Single section to show a few cells of the lateral cluster in (A). The cell marked with the yellow asterisk is an example of a GABA-ergic cell belonging to the lateral cluster, but not labelled by GAL4-*GH146*. Cells such as these are likely to be LNs. The pink asterisk represents a cell that is GAL4-*GH146 *positive and does not stain for GABA and is identified as a PN. Scale bars, 10 μm. Genotype: *Tub*-LexA::GAD/+ or Y; FRTG13, hsFLP, *Tub*-GAL80/FRTG13, UAS-mCD8, GAL4-*GH146*; LexAop-rCD2::GFP/+.

As expected for this lineage, the *tub*-LexA::GAD-driven marker labelled an entire Nb clone with cell bodies located lateral to the antennal lobe. The intense labelling of the entire antennal lobe neuropile indicates that many of these cells extend numerous processes into the glomeruli as mentioned above in the LN2 dual MARCM experiment. The *GH146*-driven marker labelled only a subset of cells in the Nb clone corresponding to approximately one-fifth of the overall lNb lineage (Figure 3A). These labelled PNs had cell bodies that were clustered together in a more ventral location of the Nb clone (red in Figure 3A4) and their dendritic processes ramified in only a subset of the antennal glomeruli (Figure 3A2). This type of glomerulus-restricted innervation is expected for the ensemble of *GH146*-labelled uniglomerular lPNs. However, it differs markedly from the type of multiglomerular labelling observed in the corresponding *tubulin*-labelled neuroblast clone, which can only be explained if many multiglomerular neurons such as the LNs, or the multiglomerular PNs described by Lai and his colleagues [26], are also present in the lineage.

**Figure 4:**
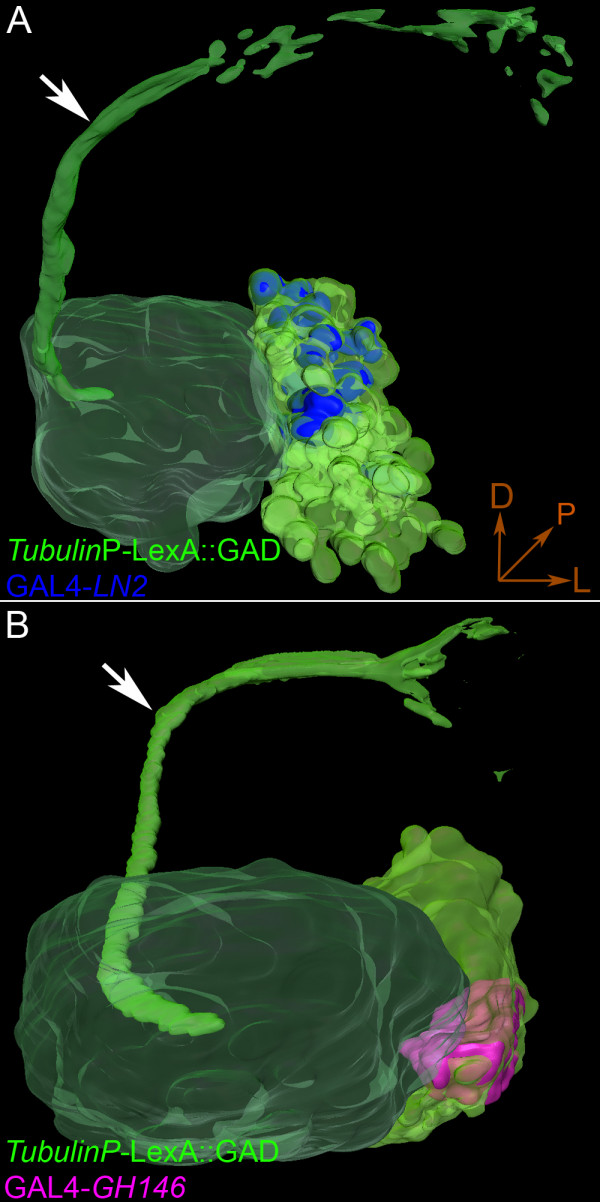
Three-dimensional reconstructions of lateral neuroblast (lNb) clones. **(A) **Reconstruction of the *LN2*-dual MARCM brain shown in Figure 2. The green cluster of cells represents the *Tub*-LexA::GAD; LexAop-rCD2::GFP marked lateral cluster clone containing projection neurons (PNs; evident from the green higher centre projection, inner antennocerebral tract (iACT), indicated by the white arrow). The GAL4-*LN2 *driven CD8 marked cells shown in blue are included within the cluster. Note that they seem to be clustered dorsally within the lateral cluster. The lobe is shaded green. **(B) **Reconstruction of the *GH146*-dual MARCM lobe. The entire lateral cluster is shown in bright green, and the GAL4-*GH146 *cells are shown in pink. The higher centre projection, iACT (green), is indicated by the white arrow. Note that GH146-PNs seem to be clustered within the clone with LN2 cells located more dorsal and unmarked cells located more ventrally. D, dorsal; P, posterior; L, lateral.

A comparison of three-dimensional reconstructed models of *LN2*-dual MARCM and *GH146*-dual MARCM experiments underscores the fact that the lNb lineage indeed gives birth to both LNs and PNs (Figure 4A,B). The *GH146*-positive uniglomerular PNs have their cell bodies clustered together in a compact group within the lNb lineage (pink in Figure 4B) while the remaining larger group of cell bodies within the lineage are *GH146*-negative. Among this large set of *GH146*-negative cells are the cell bodies belonging to the GABA-positive LNs, which are clustered together in a spatially distinct dorsal part of the lineage (Figure 3A3, blue in Figure 4A).

Taken together, these data indicate that LNs and lPNs do indeed derive from the same lNb and are thus lineage related.

### LNs do not arise from the adNb lineage

The experiments described above indicate that the lateral lineage comprises PNs and a sizeable number of LNs. Do any of the other two Nb lineages that generate PNs, the adNb and vNb, also produce LNs? To investigate this, we again performed dual expression-control MARCM experiments in which GAL4-*GH146 *and *tub-LexA*-GAD were used as drivers in order to differentially label PNs together with all cells in Nb clones. In these experiments we restricted our analysis to the double-labelled clones corresponding to the adNb and vNb lineages.

**Figure 5:**
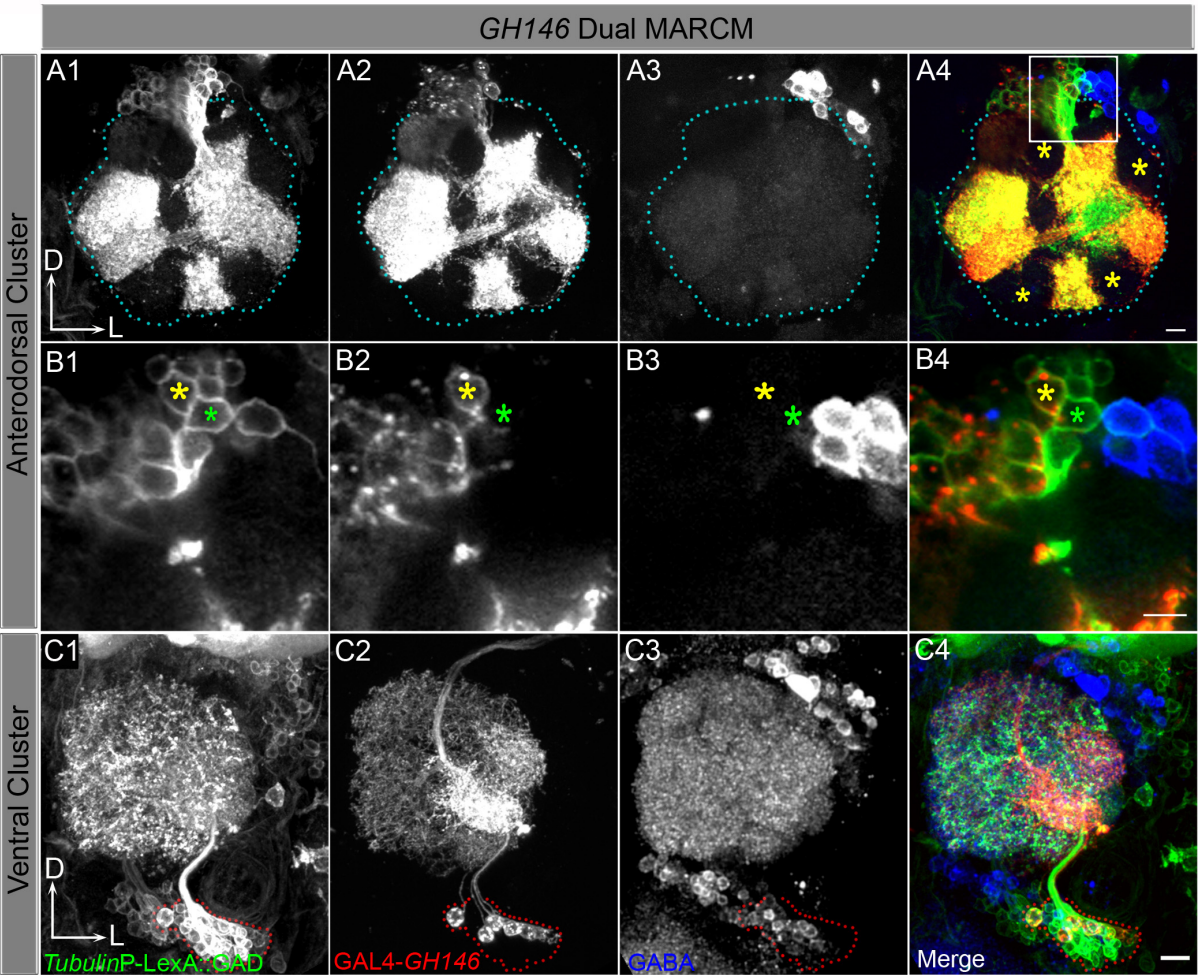
Local interneuron (LNs) do not arise from the anterodorsal cluster. **(A) **Merge of a few z-sections of the anterodorsal neuroblast clone generated at 0–4 h after larval hatching to show that LNs do not arise from this cluster. L, lateral; D, dorsal. The yellow asterisks in (A4) show that many glomeruli do not have projections from the anterodorsal cells; these cells are therefore unlikely to be LNs. **(B) **Higher magnification of a single section of a few cells from (A). All cells of this cluster, either GAL4-*GH146*-labelled or not, do not stain positive with anti-GABA (yellow asterisk and green asterisk, respectively). **(C) **Ventral cluster of second order olfactory neurons (red dotted line) containing multiglomerular projection neurons; 6–8 of them are marked by GAL4-*GH146 *(C2) as well as many GABA-ergic cells (C3). (C4) Merge. Scale bars, 10 μm. Genotype: *Tub*-LexA::GAD/+ or Y; FRTG13, hsFLP, *Tub*-GAL80/FRTG13, UAS-mCD8, GAL4-*GH146*; LexAop-rCD2::GFP/+.

In the adNb lineage, the *tub*-LexA::GAD-driven marker labelled the entire clone consisting of approximately 60–70 cells that have their cell bodies clustered anterodorsal to the antennal lobe (Figure 5). In all the Nb clones of the anterodorsal cluster (N = 12), the labelled cells projected processes into specific regions of the antennal lobe but did not cover the entire lobe (Figure 5A4, yellow asterisks). The *GH146*-driven marker labelled a large subset of the cells in the adNb clone, all of which had the expected features of uniglomerular PNs with axons projecting towards the mushroom bodies and lateral horns. Like these *GH146*-labelled PNs (Figure 5A2), the dendrites of the *tubulin*-labelled cells of the entire adNb clone were restricted to specific antennal lobe regions and never extended to innervate the entire lobe (Figure 5A1,A4). Although not all of the *tubulin*-labelled cells in the adNb clone were co-labelled by GAL4-*GH146*, these remaining *GH146*-negative cells are also likely to be PNs given their restricted glomerular innervation pattern and their axonal projections. These findings imply that there are no multiglomerular LNs within the adNb lineage. The observation that none of the cells in the adNb lineage were GABA-immunoreactive, in contrast to the lNb lineage, where many of the LNs (but none of the PNs) were GABA-immunoreactive, lends further support to this conclusion (compare Figures 5B3 and 3B3).

In the vNb lineage, the cells labelled by the *tub*-LexA::GAD driver did have processes that arborised throughout the antennal lobe (Figure 5C1). This was also the case for the small subset of these cells labelled by the GAL4-*GH146 *driver (Figure 5C2). This is in accordance with the fact that many of the PNs in the ventral cluster have multiglomerular dendritic arbors [9,15]. The cell cluster seen in Figure 5C, closely apposed to the ventral cluster (demarcated with red dots), does not project to the antennal lobe and is likely to be of a distinct lineage.

A number of the *tubulin*-positive cells in the vNb lineage were also positive for GABA immunoreactivity (Figure 5C3), consistent with previous reports that several PNs in the ventral cluster are GABA-ergic [22,25]. Though our data do not exclude the possibility that some LNs could still arise from the ventral lineage, single-cell clonal analysis by Lai and colleagues [26] indicates that all cells of this cluster are PNs.

### Lineage and birth order of LNs and PNs arising from the lNb

The data presented in the previous sections imply that the lNb gives rise to both LNs and uniglomerular PNs. Given the striking differences in morphology, connectivity as well as neurotransmitter phenotypes between LNs and PNs, we wondered if these two cell types are generated sequentially or simultaneously by this Nb. To investigate the spatial and temporal aspects of lineage relationships between LNs and PNs, we used dual expression control MARCM techniques (involving either GAL4-*GH146 *or GAL4-*LN2 *drivers along with *tub*-LexA::GAD for lineage identification) to generate single cell and double cell clones in the lNb lineage at different times during development [28,32]. Mitotic recombination was induced randomly in late embryo, or at 0–4 h, 24 h, 48 h, 72 h and 96 h ALH. The numbers of labelled cells recovered in the adult are summarised in Table 1.

**Table 1:** Numbers of clones generated at different time-points during larval life

	Embryonic	0–4 h ALH	24 h ALH	48 h ALH	72 h ALH	96 h ALH
Nb clones	11	19	12	0	1	0
Uniglomerular PNs	0	0	0	20	18	5
Multiglomerular PNs	0	0	0	1	0	0
LNs	3	19	24	31	24	9

The top row summarizes the number of clones that encompass the entire progeny of a neuroblast (Nb). The numbers of uniglomerular lateral projection neurons (lPNs), multiglomerular PNs and local interneuron (LNs) labelled at different times of clone induction. ALH, after larval hatching.

**Figure 6:**
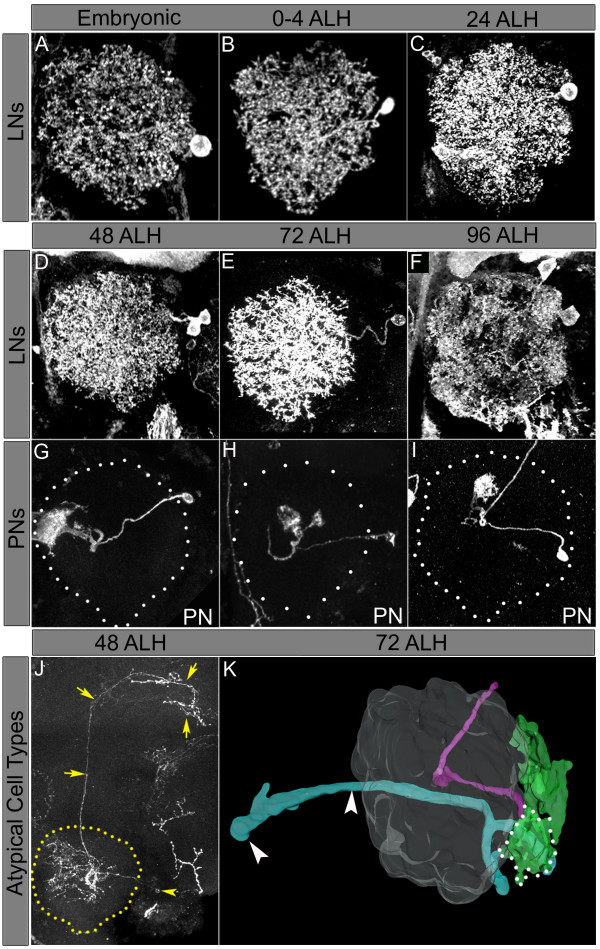
Lineage and birth-order of the local interneurons (LNs) and projection neurons (PNs) arising from the lateral neuroblast (lNb). Clones were generated at the times indicated. **(A-I) **Note that the multiglomerular LNs are born throughout larval life (A-F) and the uniglomerular lPNs appear only from 48 h after larval hatching (ALH) (G-I). **(J) **A single-cell clone of a PN with oligoglomerular projections. The cell body is marked with a yellow arrowhead and projections to the higher brain centre are marked with yellow arrows. **(K) **Three-dimensional reconstruction of a multiple cell clone generated at 72 h ALH showing cell bodies of previously undescribed PNs (green cell bodies shown within the white dotted lines), which send neurites to the antennal lobe (not shown in the reconstruction) and some tracts project out of the lobe to non-olfactory neuropiles (light blue axonal tracts), ipsilaterally as well as contralaterally (white arrowheads). PN projections from this clone are indicated in pink.

When the recombination event was performed before 48 h ALH, a set of single/double cell clones consisting of LNs was recovered (n = 46 single/double cell clones; Figure 6A–C). These LNs could be identified based on their morphology and/or GAL4-*LN2 *expression in single and double cell LN clones. The labelled cell bodies of these multiglomerular LNs were located lateral to the antennal lobe. In contrast, uniglomerular lPNs and/or cells labelled with GAL4-*GH146 *were not recovered in clones generated during these early proliferative phases. However, single-cell and double-cell lPNs with uniglomerular projections were recovered when the recombination event was performed at 48 h, 72 h and 96 h ALH (n = 44 PNs out of 108 labelled cells; Figure 6G–I). LNs also continue to be generated during later proliferative stages, and both single-cell and double-cell LN clones were observed at 48 h, 72 h and 96 h ALH (n = 64 LNs out of 108 labelled cells; Figure 6D–F).

The lNb is known to give rise to PNs other than typical uniglomerular PNs. However, multiglomerular PNs arising early in the lineage, as described by Lai and colleagues [26], were not observed in our study, perhaps because of a lack of appropriate labels for these cells. Hence, while our results suggest that the majority of cells born in the early proliferative period of the lNb might be LNs, it is likely that multi-glomerular PNs not labelled by GAL4-*GH146 *were missed in this analysis. However, we did observe single PNs with oligoglomerular projections (not labelled by GAL4-*GH146*) when clones were induced at 48 h ALH (Figure 6J). Moreover, careful analysis of labelled Nb clones suggests that there are indeed additional cells in the lateral cluster, as shown previously by Lai *et al*. [26], which send projections to antennal lobe as well as to unidentified non-olfactory neuropiles both ipsilaterally and contralaterally (reconstructed three-dimensional model in Figure 6K).

The early proliferative divisions of the lateral neuroblast (embryo to approximately 24 h ALH) that give rise to LNs are likely to occur according to the canonical division mode, in which the Nb divides asymmetrically to self renew and produce a ganglion mother cell that divides only once to produce two neurons. Correspondingly, we only observed Nb clones or single and two cell LN clones when recombination occurred before 48 h. This may also be the case for some LNs and lPNs generated during the second, later phase of proliferation, since we recovered single-cell and two-cell LN clones as well as single-cell and two-cell lPN clones when recombination was induced at 48 h, 72 h and 96 h ALH. However, during this later proliferation phase, we also recovered samples consisting of 3–6 labelled cells (Table 2). One explanation for this observation could be that there are several ganglion mother cells in the lNb lineage and that more than one of these might be competent for MARCM labelling at the time when recombination was induced [30]. In order to estimate the number of mitotically competent cells within the lNb lineage, we induced clones at 0–4 h ALH and examined the clones in the third instar larval stage after exposing the brains to 5 μg/ml bromodeoxyuridine (BrdU) for 1 h. Labelled clones of the lNb contained 5–6 BrdU positive cells, comparable to the numbers of BrdU positive cells observed in mushroom body Nb clones (data not shown). This may be indicative of an elevated proliferation rate in this neuroblast, which could lead to an accumulation of mitotically competent ganglion mother cells in the lineage. However, we cannot rule out that the observed 'atypical' multicellular clones are due to proliferative processes that cannot be adequately analysed by current genetic techniques.

**Table 2:** Clones generated in the later phase of proliferation

Clone induced 48 h ALH	Clone induced at 72 h ALH	Clone induced at 96 h ALH
1 LN, 1 PN (n = 2)	2 LN, 1 PN (n = 4)	1 LN, 2 PN (n = 1)
1 LN, 2 PN (n = 2)	3 LN, 0 PN (n = 4)	2 LN, 2 PN (n = 1)
2 LN, 2 PN (n = 1)	3 LN, 1 PN (n = 1)	3 LN, 0 PN (n = 2)
3 LN, 1 PN (n = 1)	1 LN, 2 PN (n = 1)	
5 LN, 1 PN (n = 1)	1 LN, 3 PN (n = 2)	
6 LN, 0 PN (n = 1)	5 LN, 0 PN (n = 1)	
	6 LN, 0 PN (n = 1)	

Cellular compositions of 26 clones, possibly generated from multiple recombination events, obtained from a total of 197 clonal antennal lobes are represented. The time points of heat shocks are given in the first row and the number of occurrences of each type of clone is given in brackets. ALH, after larval hatching; LN, local interneuron; PN, projection neuron.

Taken together, these findings argue for two distinct proliferative phases in the lNb lineage – an early phase in which LNs but no uniglomerular lPNs are generated and a later phase in which both LNs and lPNs are formed. This suggests that the lNb undergoes an alteration in its proliferation competence between 24 h and 48 h ALH with respect to the neuron types generated.

### LNs are a morphologically diverse population of neurons

As noted above, the population of LNs that derive from the lNb is diverse in its neurotransmitter phenotype and consists of GABA-ergic and cholinergic neurons, and possibly other neurotransmitter types. These LNs also manifest a surprisingly diverse set of neuronal morphologies as revealed by single-cell MARCM clones. Our findings show that many LNs uniformly innervate the entire antennal lobe. However, in contrast to earlier assumptions, we also find many other LNs that have a more restricted innervation pattern.

**Figure 7:**
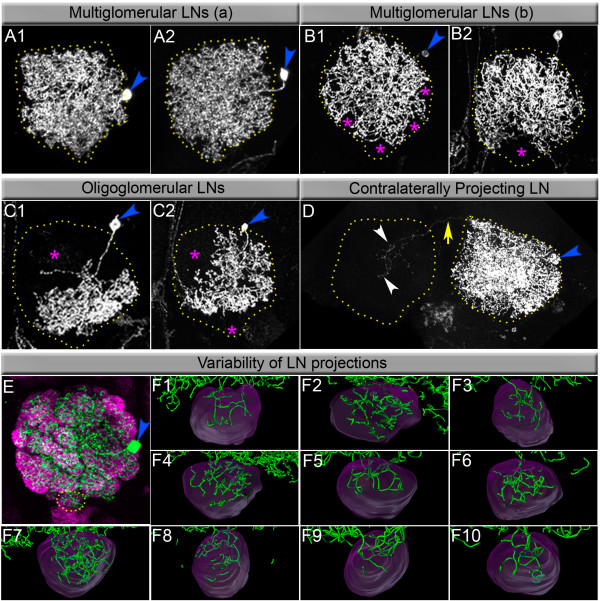
Heterogeneity of local interneurons (LNs). Single cell clones are heterogeneous in their patterns of arborization. **(A, B) **Multiglomerular neurons innervate several glomeruli. Type a (A1, A2) innervates a large fraction while type b (B1, B2) cells have somewhat more restricted innervation. Pink asterisks highlight glomeruli that are excluded from the field of this LN. **(C) **Oligoglomerular LNs innervate a smaller subset of glomeruli. **(D) **Contralaterally projecting LNs send at least one branch to the other antennal lobe through commissure (yellow arrow), which branches and innervates some glomeruli (white arrowheads). Cell bodies in each case are indicated by blue arrowheads. **(E) **A representative GAL4-*LN2 *MARCM single cell clone generated at 0–4 h after larval hatching (ALH). **(F) **Three-dimensional reconstructions of 10 single cell LN clones born 0–4 h ALH and focusing on their innervations in the V glomerulus.

To document this morphological diversity, we carried out a detailed examination of the dendritic projections of 76 labelled LNs in the antennal lobe. Several different types of LNs were found. Figure 7A shows examples of LNs that innervate the entire ipsilateral antennal lobe without a distinct glomerular innervation pattern. These correspond to the multiglomerular-type A LNs [22,26]. Figure 7B shows examples of LNs that innervate large regions of the ipsilateral antennal lobe but their processes exclude specific glomerular regions. These may correspond to mutliglomerular-type B LNs [22]. A third type of LN is oligoglomerular and has a more restricted innervation pattern. As shown in figure 7C, LNs of this type innervate only a part of the ipsilateral antennal lobe and, correspondingly, project to only a subset of the olfactory glomeruli. Finally, in figure 7D, we show an example of the LN type that innervates the ipsilateral antennal lobe and, in addition, sends processes into the contralateral antennal lobe.

In this study, we were not able to individually identify a given LN and investigate its morphology in different individuals. We were therefore not able to determine the degree of anatomical variability in the dendritic projection patterns of an individual LN with precision. In order to estimate the variability of glomerular innervation, we selected 10 single-cell LN clones generated by heat-shock between 0 and 4 h ALH and analysed the branching patterns of these individual neurons within the easily identifiable glomerulus-V (Figure 7E,F). These experiments suggest that the innervation of a given glomerulus by LNs born during a similar short time interval does show considerable differences. More rigorous analysis of this putative variability must, however, await techniques for the reliable identification of individual LNs.

### The empty spiracles gene is required for LN development

The cephalic gap gene *empty spiracles *(*ems*) is required for embryonic development of the antennal brain neuromere and is also essential for correct PN development in postembryonic stages [33,34]. In the PNs from the adNb lineage, *ems *is necessary for precise targeting of PN dendrites to appropriate glomeruli [33]. In the PNs of the lNb lineage, *ems *is required for the development of the correct number of PNs; in *ems *mutants, the number of neurons in this lineage is markedly reduced. To determine if *ems *also plays a role in postembryonic development of LNs, wild-type and *ems *mutant MARCM clones were generated. Clones were induced at random in the early first instar and analysed in the adult; LNs were labelled by GAL4-*LN1 *or GAL4-*LN2 *driving UAS-mCD8::GFP.

**Figure 8:**
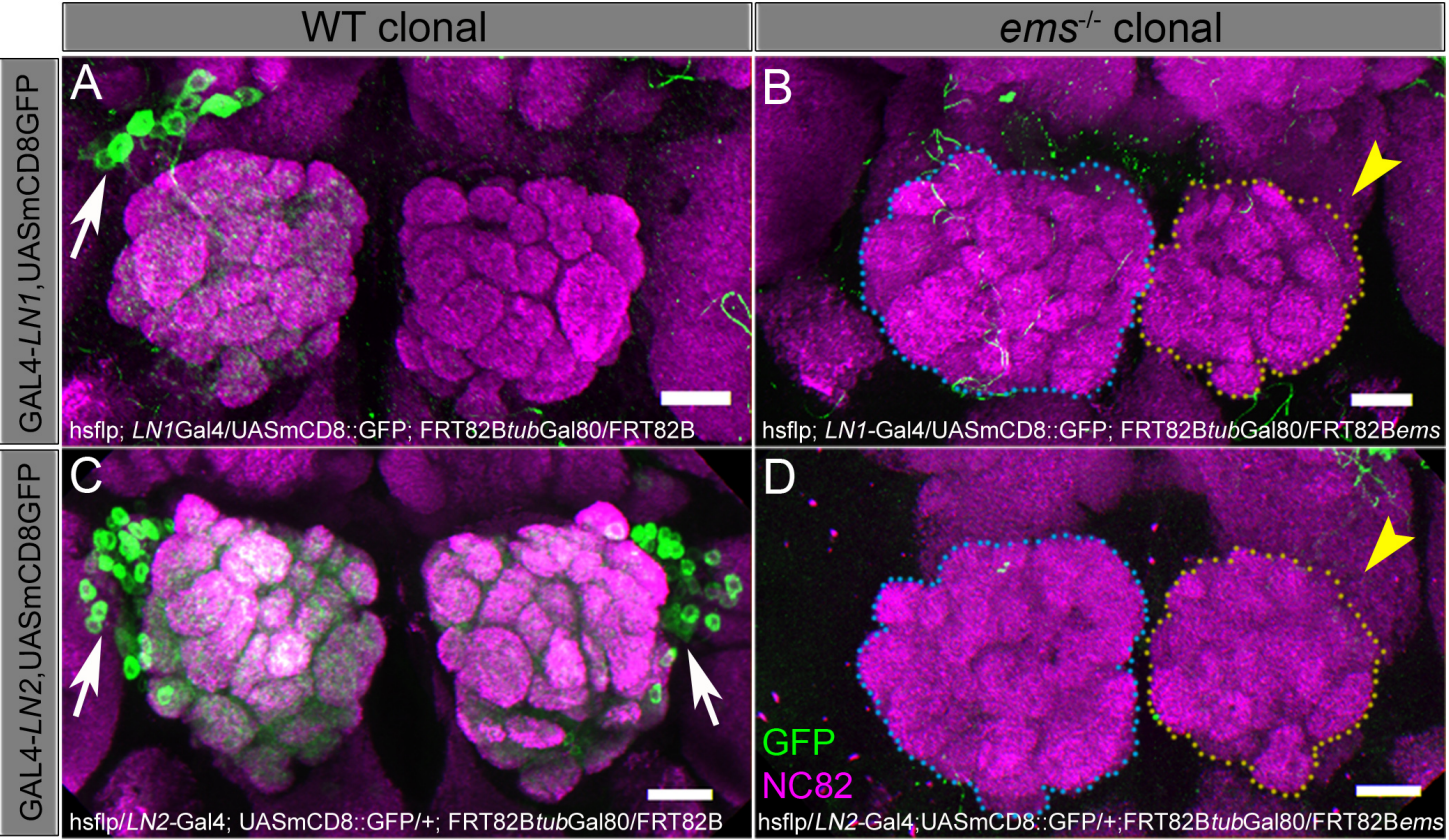
*ems *loss of function leads to a loss of neurons derived from the lateral neuroblast (lNb). **(A, C) **Wild-type MARCM clones of GAL4-*LN1*, UAS-mCD8::GFP and GAL4-*LN2*, UAS-mCD8::GFP generated between 0–4 h after larval hatching. White arrows indicate Nb clones. **(B, D) ***em*s mutant clonal brains. Note that no cells are visible although the shrunk lobe (encircled by yellow dots) indicates that clones have formed and have been ablated due to loss of *ems *(yellow arrowheads). Scale bars, 20 μm. Genotype in (A): *y*, *w*, hsFLP/+ or Y; GAL4-*LN1*/UAS-mCD8::GFP; FRT82B *tub*-GAL80/FRT82B. Genotype in (B): *y*, *w*, hsFLP/+ or Y; GAL4-*LN1*/UAS-mCD8::GFP; FRT82B *tub-GAL80*/FRT82B, *ems*^9*Q*64^. Genotype in (C): *y*, *w*, hsFLP/GAL4-*LN2*; UAS-mCD8::GFP/+; FRT82B *tub*-GAL80/FRT82B. Genotype in (D): *y*, *w*, hsFLP/GAL4-*LN2*; UAS-mCD8::GFP/+; FRT82B *tub*-GAL80/FRT82B, *ems*^9*Q*64^.

In wild-type controls, cells labelled by GAL4-*LN1 *or GAL4-*LN2 *were often observed. Among 83 brains (166 lobes) examined, 49 (30% of the antennal lobes) had labelled wild-type clones comprising LNs, of which approximately 10% were Nb clones. As expected, these labelled LNs had cell bodies in the lateral/dorsolateral region of the antennal lobe and processes that ramified extensively within multiple glomeruli (Figure 8A,C). In contrast, in the *ems *mutant MARCM experiments, labelled Nb clones were never observed. Among 55 brains (110 lobes) examined, none had labelled *ems *mutant multiple cell LN clones in the lateral cluster. This suggests that *ems *mutation leads to a lack of LNs in the lNb lineage. In accordance with this assumption, a marked reduction in size of the antennal lobes in the *ems *mutant MARCM experiments was often observed. Thus, among the 55 brains examined, 15 had a marked reduction in one antennal lobe and 4 had a reduction in both antennal lobes (Figure 8B,D). We confirmed that the phenotype was due to the *ems *mutation by driving UAS-*ems *in *ems*^-/- ^mutant *tubulin *MARCM clones. The lateral cluster neurons were completely rescued in five out of six Nb clones analysed (data not shown).

Taken together, these experiments indicate that *ems *is required for the development of the lNb lineage. The observed absence of LNs in *ems *mutant clones implies that these cells either are not generated or die during postembryonic development. Lichtneckert *et al*. [35] expressed the pancaspase inhibitor P35 to demonstrate that postmitotic cell death in the absence of *ems *is responsible for the phenotype in the lNb cluster. When apoptosis was blocked in *tubulin ems *null clones, there was a partial rescue of the phenotype in third instar larvae. We confirmed that the rescue obtained upon P35 ectopic expression extended to the *ems*^-/- ^LNs within *tubulin *MARCM clones in adults (data not shown).

## Discussion

### Lineage-specific development of LNs

The LNs of the *Drosophila *antennal lobe are likely to derive from a single identified neuroblast lineage, namely the lNb lineage. A number of findings support this notion. First, earlier work involving HU-mediated Nb ablation indicates that a group of approximately 20 LNs marked by GAL4-*GH298 *derives from the lNb [20]. Second, experiments combining HU-mediated Nb ablation with GAL4-*LN1 *and GAL4-*LN2 *labelling reveal that a set of approximately 60 LNs also derives from the lNb. Third, dual expression-control MARCM experiments involving GAL4-*LN2 *labelled LNs or GAL4-*GH146 *labelled lPNs indicate that both labelled cell types belong to the same lNb lineage. Fourth, dual expression-control MARCM experiments show that GAL4-*GH298 *labelled LNs, GAL4-*146 *labelled lPNs as well as oligoglomerular PNs and cells with complex architecture labelled by Acj6-GAL4 belong to the same lineage [26]. In contrast to the lNb, the adNb does not appear to generate LNs. Rather, this Nb seems to produce a lineage that is dedicated to PNs [9,16,20,32,36]. While we cannot rule out that the vNb, nor any other, currently uncharacterised Nbs located in the antennal lobe region, contribute some LNs to the olfactory circuitry, we posit that most LNs are lineage related and derive from the same Nb.

The lNb is comparable to the four Nbs that give rise to the mushroom body in that it initiates proliferation in the embryo and continues to proliferate without a quiescent phase throughout larval development [30,37]. Due to this prolonged proliferative phase, the lNb can generate an unusually large number of neuronal progeny. At late third larval instar stages, lNb clones contain approximately 200 neurons [33], which are largely conserved in the adult. The finding that a substantial proportion of the olfactory interneurons present in the adult brain, namely a majority of the LNs and a large percentage of PNs, derive from the same lNb lineage underscores this fact and highlights the role of the lNb in producing an ensemble of neurons with important roles in olfactory processing.

### Differences in birth order of LNs and lPNs

Given that LNs and PNs can be generated by the same Nb, it is interesting that uniglomerular lPNs are only generated in the later proliferative phase of the lNb. Single-cell and double-cell MARCM clones induced in the lNb lineage in the embryo or early larval stages (0–24 h ALH) were composed of LNs, and Lai and colleagues [26] described the birth of diverse atypical PNs during this period [26]. However, lPNs were only recovered if the clones were induced at 48 h or later. In other cases in which the neuron types generated by identified Nbs have been characterised, early born neurons are usually projection interneurons or motoneurons, which often pioneered central nervous system tracts or peripheral nerves, whereas local interneurons are usually among the later born neurons [38-41]. Previous work has shown that PNs target their dendrites to specific regions of the antennal lobe before the arrival of their partner ORN axons [42,43]. It is noteworthy that LNs are also present at the lobe at this time and, in the case of the lPNs, perhaps earlier. The possible role of LNs in pattering the synaptic structures in the antennal lobe has not yet been studied.

During the second larval instar stage (between 24 h and 48 h ALH), an alteration in proliferation competence appears to occur in the lNb, and production of uniglomerular lPNs is initiated along with the ongoing and continuing production of LNs in this lineage. It is noteworthy that in the adult brain, cell bodies of the early born LNs were markedly larger in size than those of the lPNs (compare cells marked with pink and yellow asterisks in Figure 3B). Previous reports have demonstrated that in several Nb lineages, the early born neurons are significantly larger in size than their later born siblings, and it will now be important to determine if the temporal series of transcription factors regulates this and other key events in LN and lPN development in the lNb lineage [44].

### Morphological diversity of LNs

LNs are important elements in the olfactory system; they interconnect glomeruli in the antennal lobe and have specific roles in modifying the information flow between ORNs and PNs [22-24,45]. An appreciation of their morphological complexity and diversity can be attained by using GAL4 lines to selectively label these neurons either as populations or, in combination with MARCM techniques, as single-cell and double-cell clones. When large populations of LNs are targeted, these labelling techniques show that ensembles of LNs establish dense dendritic arborisations throughout the antennal lobes.

When individual LNs are labelled, complex arbors throughout the olfactory glomeruli are also observed in many cases, underlining the multiglomerular nature of specific LNs. However, labelling of individual neurons also clearly reveals a hitherto unexpected degree of morphological diversity of LNs. Thus, careful examination of the extent of the dendritic arbors of single LNs shows that there are at least two different types of multiglomerular LNs. Moreover, many examples of oligoglomerular LNs manifesting different degrees of innervations of restricted sets of glomeruli as well as LNs with ipsilateral and contralateral innervations have now been found.

The remarkable morphological diversity of LNs, together with the fact that LNs express different neurotransmitters, implies that this cell type might play important roles in olfactory information processing that were not appreciated in earlier studies. This notion, together with the possibility that the innervation of individual LNs might be much more variable than currently assumed, will be important areas for further studies.

### Conserved roles of ems in olfactory system development

Despite the obvious difference in their morphology, LNs and PNs do share at least one important developmental genetic feature. The correct development of both cell types requires the cephalic gap gene *ems*. The *ems *gene, which encodes a homeodomain transcription factor, is known to be expressed in the anterodorsal and lateral Nbs and has cell lineage-specific functions in postembryonic PN development [33]. In the adNb lineage, *ems *expression is required for precise targeting of PN dendrites to appropriate glomeruli. In the lNb lineage, *ems *is essential for development of the correct number of PNs. The results of our experiments indicate that *ems *is also essential for the development of the correct number of LNs within this lineage. Thus, both types of olfactory interneurons in the first order olfactory centre of the *Drosophila *brain require Ems for proper development. Indeed, given that the *ems *gene is also expressed in the developing cephalic segment from which the antennal sense organs derive [46-48], the same gene might be important for the development of all three principal populations of neurons that form synapses in the antennal lobe neuropile, ORNs, PNs and LNs.

The organization of the olfactory system in insects and mammals is surprisingly similar [2,49]. ORNs that express a given odorant receptor send axons to the same glomeruli located in the first order olfactory centre of the brain (antennal lobe in insects, olfactory bulb in mammals). There, ORNs make specific synapses with the dendrites of two types of second order olfactory neurons, the local interneurons (LNs in insects, periglomerular cells in mammals) and the projection neurons (PNs in insects, mitral/tufted cells in mammals). Genes of the *ems*/*Emx *family are required for proper development of the first order olfactory centre in both insects and mammals. In *Drosophila*, *ems *loss-of-function leads to perturbations in LN and PN development and, hence, significant reduction in antennal lobe size. In the mouse, *Emx1 *and *Emx2 *double mutants have marked deficits in growth and lamination of the olfactory bulb; the mitral cell layer, external plexiform layer and glomerular layer are thin and poorly organised [50]. The strikingly similar expression and function of the *ems*/*Emx *genes in the development of the primary olfactory centres in insects and mammals argue for evolutionarily conserved roles of these gene homologues in olfactory system development.

## Conclusion

We have demonstrated that the lNb of the deutocerebrum gives rise to LNs and PNs that contribute to the olfactory circuit of *Drosophila*. Moreover, we have shown that LNs display a remarkable morphological diversity. LN formation is initiated early in the life of the lNb, while uniglomerular PNs are detected only after approximately 48 h ALH. The formation of both cell types in this neuroblast lineage is determined by the function of the *ems *gene.

## Materials and methods

### Fly strains and genetics

All stocks, unless otherwise mentioned, were obtained from the Bloomington Stock Centre, Indiana, USA. All stocks used for dual expression control MARCM experiments (*y*, *w*, *tub*-LexA::GAD; *Pin/CyO, y*^+^, *y*, *w*; FRT G13, hsFLP, *tub*-GAL80/*CyO, y*^+^, *y*, *w*; FRTG13, UAS-mCD8, lexAop-rCD2::GFP/*CyO, y*^+^, *y*, *w*; FRTG13, GAL4-*GH146*, UAS-mCD8/*CyO, y*^+^, *y*, *w*; *Pin/CyO*, *y*^+^; lexAop-rCD2::GFP) were kindly provided by Tzumin Lee [32]. *Cha*::dsRed, GAL4-*KL78*, GAL4-*KL107 *and *Krasavietz*-GAL4 were obtained from Gero Meisenbock [24]. GAL4-*NP1227 *(referred to as GAL4-*LN1*) and GAL4-*NP-2426 *(also called GAL4-*LN2*) were generated by the NP consortium, Japan [25].

### MARCM and dual expression control MARCM experiments

In order to follow the lineages of the LNs and PNs, we used MARCM as well as dual expression control MARCM [28,32]. Dual expression control MARCM allows marking of clonal cells by GAL80-regulated *tub*-LexA::GAD, which drives lexAop-rCD2::GFP; while the second expression system, GAL4-*LN2 *or GAL4-*GH146*, drives UAS-mCD8. The clone was visualised by staining against green fluorescent protein (GFP) while the LNs or PNs were visualised by using an antibody against the CD8 epitope. To generate clonal animals, females of *tub*-LexA::GAD; FRT G13, hsFLP, *tub*-GAL80/*CyO, y*^+ ^were crossed to males of either GAL4-*LN2*; FRTG13, UAS-mCD8, lexAop-rCD2::GFP/*CyO*-GFP or *y*, *w*/*Y*; FRTG13, GAL4-*GH146*, UAS-mCD8/*CyO, y*^+^; lexAop-rCD2::GFP. For single MARCM experiments females of *y*, *w*, hsFLP; *tubulin*-GAL4, UAS-mCD8::GFP; FRT82B, *tub*-GAL80 and *y*, *w*, hsFLP; GAL4-*LN1/CyO*-GFP; FRT82B, *tub*-GAL80 were crossed to males of UAS-LacZ, UAS-mCD8::GFP/*CyO*-GFP; FRT82B and females of the genotype GAL4-*LN2*, UAS-mCD8::GFP; FRT82B *tub*-GAL80 were crossed to males of *y*, *w*, hsFLP/*Y*; UAS-LacZ, UAS-mCD8::GFP/*CyO*; FRT82B. Embryos from the above crosses were collected at 4 h intervals and reared at 25°C. Heat shocks were given at the required time points for 1 h in a water bath maintained at 37°C. Cultures were returned to 25°C and animals were allowed to develop to adulthood.

In order to generate clones of cells null for *ems*, females of *y*, *w*, hsFLP; GAL4-*LN1*, UAS-mCD8::GFP; FRT82B *tub*-GAL80, or *y*, *w*, hsFLP; UAS-LacZ, UAS-mCD8::GFP/*CyO*; FRT82B, *ems*^9*Q*64^/MKRS were crossed to males of FRT82B, *ems*^9*Q*64^/*TM6B *or GAL4-*LN2*, UAS-*mCD8::GFP*; FRT82B, *tub*-GAL80. For the *ems *or P35 rescue experiments, female *y*, *w*, hsFLP; *tubulin*-GAL4, UAS-mCD8::GFP; FRT82B *tub*-GAL80 flies were crossed out to males of the genotype *w*^-^; UAS-*ems/CyO*; FRT82B *ems*^9*Q*64^/*TM3 *or *w*^-^; UAS-P35/*CyO*; FRT82B, *ems*^9*Q*64^/*TM3*. Heat shocks were given at 0–4 h ALH for 1 h in a water bath maintained at 37°C and cultures were returned to 25°C for adults to emerge. In all cases, whole mount brains of adults were stained for the presence clones with anti-GFP or anti-CD8 and synaptic neuropiles were marked using an antibody against the presynaptic protein Bruchpilot (mAbnc82).

### Hydroxyurea ablation

Newly hatched larvae (0–4 h old) were collected and fed on yeast paste containing 50 mg/ml hydroxyurea for 4 h. They were washed with distilled water repeatedly and allowed to grow on regular cornmeal media until adulthood. Animals of genotype GAL4-*LN1*/UAS-mCD8::GFP or GAL4-*LN2*/+;UAS-mCD8::GFP were dissected and brains stained with anti-GFP and mAbnc82.

### Immunohistochemistry

Brains were dissected and stained as described earlier [43,51]. Primary antibodies used were: rabbit anti-GFP (1:10,000; Molecular Probes, Invitrogen, Delhi, India), chick anti-GFP (1:500; AbCam, Cambridge, UK), rat anti-mCD8 (1:100; Caltag Laboratories, Burlingame, CA, USA), mouse anti-Bruchpilot (mAbnc82, 1:20; DSHB, Iowa, USA), mouse anti-prospero (1:4; DSHB), rabbit anti-GABA (1:500; cat#A2052, Sigma, St Louis, MO, USA). Secondary antibodies – Alexa-488, Alexa-568 and Alexa-647 coupled antibodies generated in goat (Molecular Probes) – were used at 1:400 dilutions.

For BrdU incorporation *tubulin*-MARCM clones were generated at 0–4 h ALH. Third instar larval brains were dissected and incubated in 5 μg/ml BrdU solution in phosphate buffered saline for 1 h at room temperature with gentle shaking. Brains were fixed in 5% formaldehyde for 30 minutes and washed three times for 5 minutes each in 0.3% PTX (0.3% Triton X in phosphate buffered saline). They were treated with 2N HCl for 30 minutes followed by 0.1 M boric acid solution for 2 minutes. Blocking was carried out in 5% Normal Goat Serum in 0.3% PTX followed by incubation in rat anti-BrdU (1:100; Abcam) diluted in 5% Normal Goat Serum in 0.3% PTX overnight at 4°C on a shaker. After washing in 0.3% PTX for 15 minutes, brains were incubated in fluorophore coupled anti-rat secondary for 2 h at room temperature.

After extensive washing, stained preparations were mounted between two coverslips (with spacers) and imaged on an Olympus Fluoview (FV1000) or Leica TCS SP scanning confocal microscope. Data for Figure 2 were acquired using a Zeiss Apotome and BioRad Radiance 2000 confocal microscope. Optical sections were acquired at 0.75 μm intervals with a picture size of 512 × 512 pixels. Images were digitally processed using ImageJ [52] and Adobe Photoshop CS3. Three-dimensional reconstructions were generated using Amira (version 4.1 and 5.1; TGS, Merignac Cedex, France).

## Abbreviations

AdNb: anterodorsal neuroblast; ALH: after larval hatching; BrdU: bromodeoxyuridine; GFP: green fluorescent protein; HU: hydroxyurea; LN: local interneuron; lNb: lateral neuroblast; lPN: lateral PN; MARCM: mosaic analysis with a repressible cell marker; ORN: olfactory receptor neuron; PN: projection neuron; vNb: ventral neuroblast.

## Competing interests

The authors declare that they have no competing interests.

## Authors' contributions

AD and SS carried out all the experiments, RL helped in designing the genetic strategies, VR and HR conceptualised the project, and KI and RO generated and characterised the marker lines that allowed marking of the local interneurons. All authors participated in the preparation of the manuscript.

## Supplementary Material

Additional file 1 Expression pattern of P(Gal4) lines marking antennal lobe interneurons. **(A) **Expression pattern of Gal4-*GH146*, UAS-mCD8::GFP in the right antennal lobe. PN cell bodies lie in three clusters – anterodorsal (blue arrowhead), lateral (yellow arrowhead) cells and ventral (cyan arrowhead). D, dorsal; L, lateral. **(B-D) **Expression patterns of three lines – *Krasavietz*-GAL4, GAL4-*KL107 *and GAL4-*KL78 *– marking local interneurons (LNs) to show their location around the antennal lobe. Yellow arrowheads show the lateral location of LN cell bodies. Click here for file

Additional file 2 **Hydroxyurea treatment during 0–4 hours ALH ablates LNs**. **(A, C) **Wild-type GAL4-*LN1*, UAS-mCD8::GFP and GAL4-*LN2*, UAS-mCD8::GFP expression patterns, respectively. Their cell bodies are encircled by white dots. **(B, D) **GAL4-*LN1*, UAS-mCD8::GFP and GAL4-*LN2*, UAS-mCD8::GFP adults, which were fed hydroxyurea at 0–4 h after larval hatching. Note that the lobes encircled by the yellow dots are shrunk in size compared to the lobes that have contribution from all cells encircled by blue dots. Scale bars, 20 μm. Click here for file
