# Supplementary figures and images for: Drosophila olfactory local interneurons and projection neurons derive from a common neuroblast lineage specified by the empty spiracles gene
Source: Neural Dev. 2008 Dec 3;3:33. doi: 10.1186/1749-8104-3-33 (PMC2647541; doi:10.1186/1749-8104-3-33)

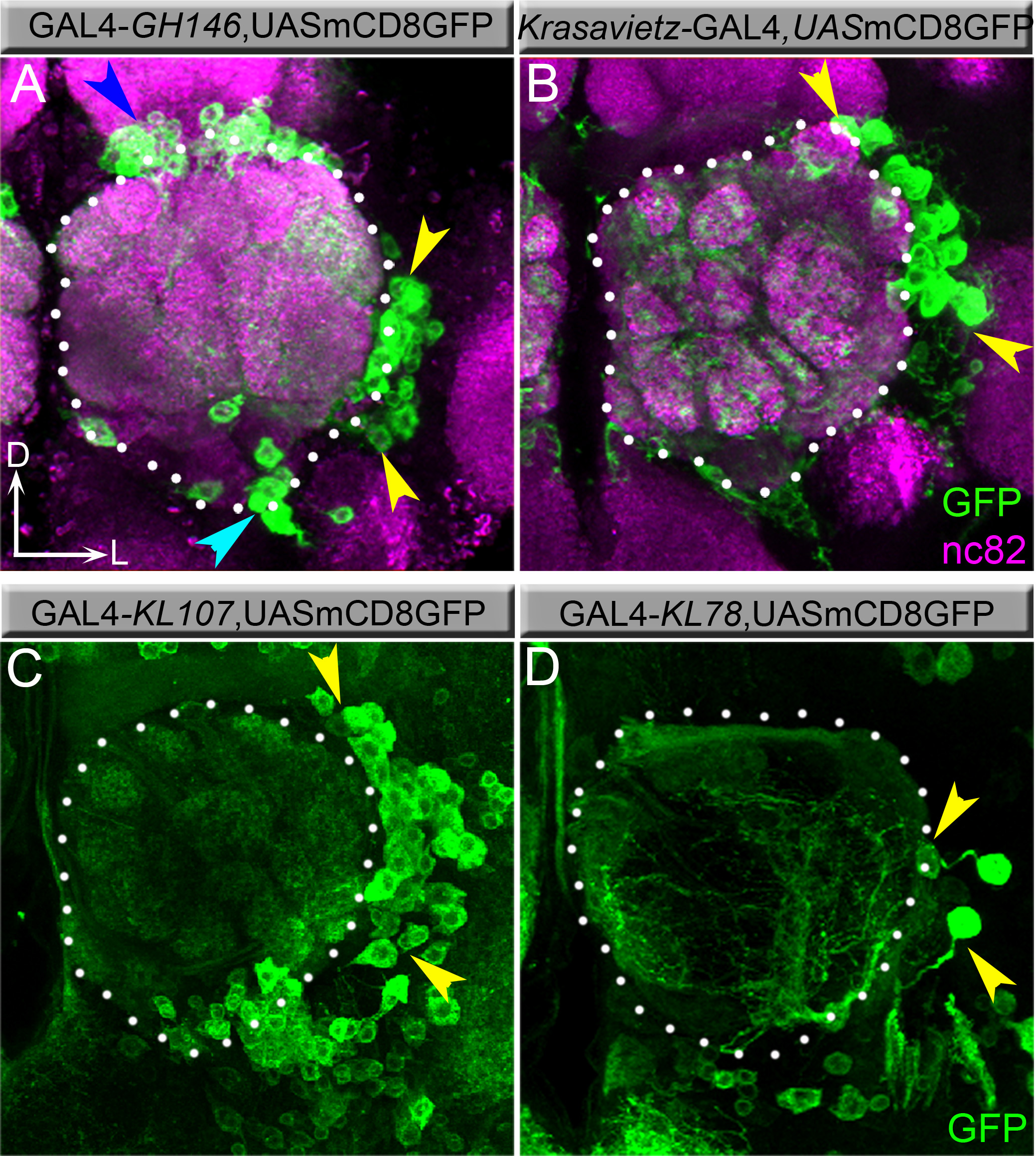

Supplement: Additional file 1 — Expression pattern of P(Gal4) lines marking antennal lobe interneurons. (A) Expression pattern of Gal4-GH146, UAS-mCD8::GFP in the right antennal lobe. PN cell bodies lie in three clusters – anterodorsal (blue arrowhead), lateral (yellow arrowhead) cells and ventral (cyan arrowhead). D, dorsal; L, lateral. (B-D) Expression patterns of three lines – Krasavietz-GAL4, GAL4-KL107 and GAL4-KL78 – marking local interneurons (LNs) to show their location around the antennal lobe. Yellow arrowheads show the lateral location of LN cell bodies. [file 1749-8104-3-33-S1.jpeg]

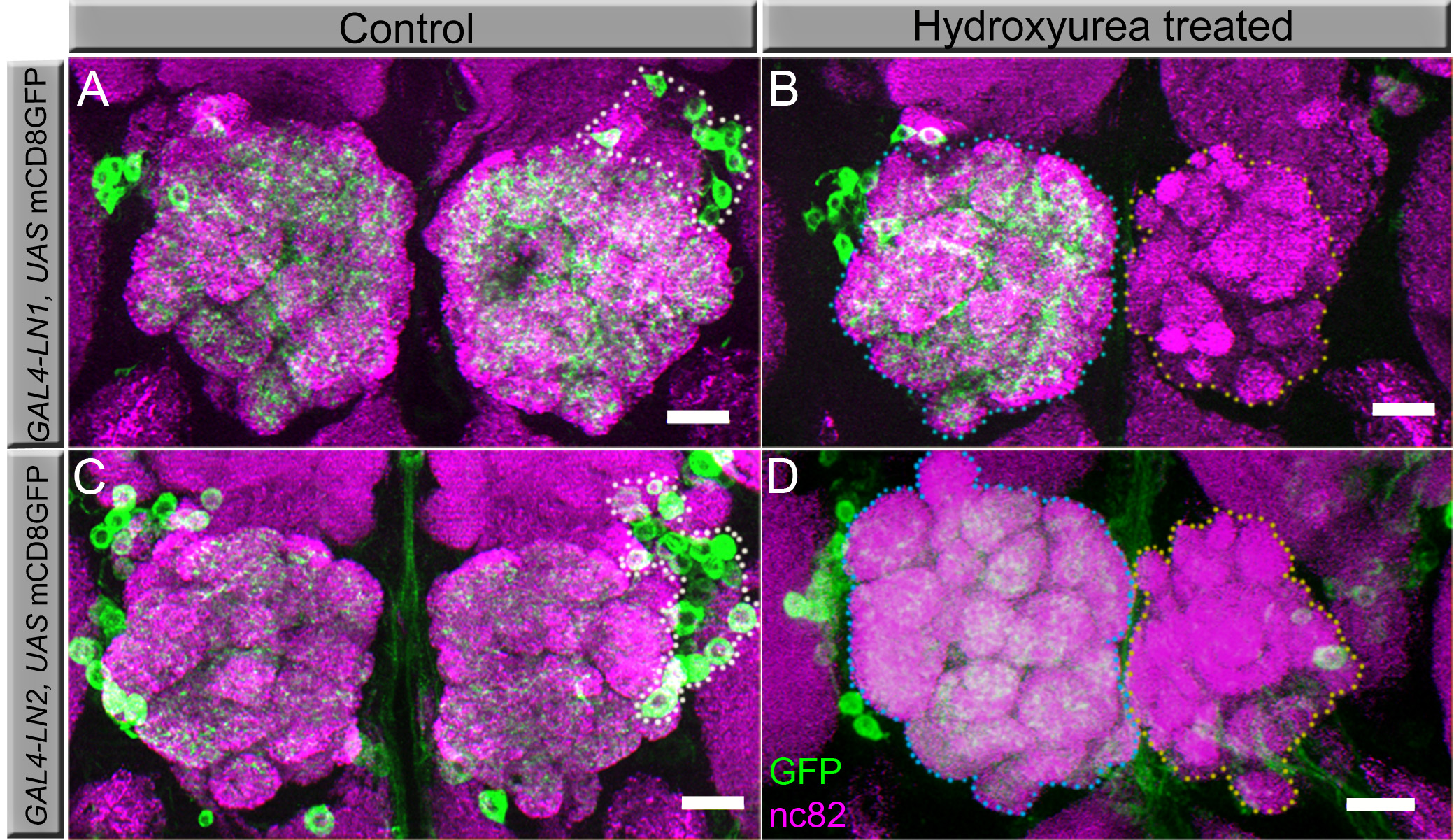

Supplement: Additional file 2 — Hydroxyurea treatment during 0–4 hours ALH ablates LNs. (A, C) Wild-type GAL4-LN1, UAS-mCD8::GFP and GAL4-LN2, UAS-mCD8::GFP expression patterns, respectively. Their cell bodies are encircled by white dots. (B, D) GAL4-LN1, UAS-mCD8::GFP and GAL4-LN2, UAS-mCD8::GFP adults, which were fed hydroxyurea at 0–4 h after larval hatching. Note that the lobes encircled by the yellow dots are shrunk in size compared to the lobes that have contribution from all cells encircled by blue dots. Scale bars, 20 μm. [file 1749-8104-3-33-S2.jpeg]
